# Perceived social fairness and trust in government serially mediate the effect of governance quality on subjective well-being

**DOI:** 10.1038/s41598-024-67124-4

**Published:** 2024-07-10

**Authors:** Yongqiang Ma, Baobin Ma, Lichun Yu, Mingyang Ma, Yibing Dong

**Affiliations:** 1 School of Marxism, Mudanjiang Normal University, Mudanjiang, 157011 Heilongjiang China; 2 School of Urban Economics and Public Administration, Capital University of Economics and Business, Beijing, 100070 China; 3 Academic Exchange Department, Party School of the Heilongjiang Provincial Committee of the Communist Party of China, Harbin, 150080 Heilongjiang China

**Keywords:** Environmental social sciences, Environmental impact, Socioeconomic scenarios, Sustainability

## Abstract

Governance quality refers to how well the processes and institutions of public governance function and is widely recognized as having an important influence on human well-being. We developed and tested a theoretical model that elucidates the relationship between governance quality and the subjective well-being of residents in China by revealing the serial mediation effects of perceived social fairness and trust in government. Using data from the nationally representative Chinese Social Survey conducted in 2021 (*n* = 5019), we performed structural equation modeling to empirically examine our hypotheses. The results indicated that governance quality exerted a significant positive fully indirect impact on subjective well-being through perceived social fairness, trust in government, and their serial mediation effects. This study contributes to the literature by providing valuable insights into the determinants of subjective well-being and highlighting the serial mediating roles of perceived social fairness and trust in government in the relationship between governance quality and subjective well-being. The findings also provide practical insights for policymakers, as they indicate that promoting perceived social fairness and fostering trust in government are essential to translate governance quality into subjective well-being.

## Introduction

Subjective well-being, also known as happiness or life satisfaction, has been recognized as a fundamental value and goal of humans since ancient times^1^. Studies have shown that individual factors such as personality, emotions, physical and mental health, and satisfaction of basic needs, as well as societal factors such as the institutional and cultural environment, predict changes in subjective well-being^2^. Recent research has highlighted governance quality as an important factor in individuals’ subjective well-being, acknowledging its multifaceted impact on societal functioning and public administration^3–6^. Governance quality encompasses the effectiveness, transparency, accountability, and fairness of governing institutions and processes^7^. It influences not only the provision of public services and the rule of law but also the broader social and economic conditions that contribute to people’s subjective well-being. Understanding the mechanisms through which governance quality influences subjective well-being is therefore important in promoting societal harmony and individual happiness.

While governance quality and trust in government are highly related concepts, they differ in their focus and implications. Governance quality encompasses broader aspects, including transparency, accountability, and effectiveness in delivering public services. It relates to the overall institutional framework that shapes citizens’ experiences and expectations. In contrast, trust in government specifically pertains to people’s trust and confidence in the government as an entity. Trust in government reflects perceptions of the government’s reliability, competence, and responsiveness to the needs and concerns of its citizens. Research suggests that governance quality influences subjective well-being through multiple channels. One such channel is the impact of governance quality on individuals’ perceived social fairness, which reflects their beliefs about the equitable distribution of resources^8^. High-quality governance fosters a sense of justice and inclusiveness that enhances subjective well-being^9–11^. Another important mechanism connecting governance quality and subjective well-being is trust in government, which signifies people’s confidence in competent and responsive authorities^12^. High-quality governance promotes trust in government, fostering security, cooperation, and civic engagement, which contribute positively to subjective well-being^13–15^. However, previous studies have primarily conducted either simple correlations between governance quality and subjective well-being or simplistic single mediator models^4–6,15–19^. These approaches have failed to fully capture the complex mechanisms underlying the association between governance quality and subjective well-being. Consequently, there is a lack of exploration of multiple complex models that could provide a deeper understanding of the potential mediating variables that link governance quality and subjective well-being. In particular, although previous research has established a positive relationship between perceived social fairness and trust in government^20^, the potential sequential mediation effects of these factors on the relationship between governance quality and subjective well-being have not been investigated.

Therefore, the main objective of this study was to develop a theoretical framework that explains the link between governance quality and subjective well-being through the serial mediation effects of perceived social fairness and trust in government. Accordingly, we aimed to answer the following questions: How does governance quality influence residents’ subjective well-being in China? Do perceived social fairness and trust in government play a serial mediating role in the relationship between governance quality and subjective well-being? To achieve this, we used nationally representative data from the 2021 Chinese Social Survey (CSS) to investigate the serial mediation effects of perceived social fairness and trust in government on the association between governance quality and subjective well-being.

This study makes several noteworthy contributions to the literature. Specifically, it elucidates the sequential multiple mediating effects of perceived social fairness and trust in government on the relationship between governance quality and subjective well-being. The use of rigorous statistical analysis techniques enhances the validity of the findings and strengthens the confidence in the conclusions drawn. Furthermore, the findings enhance our understanding of the potential of governance quality as a key factor in improving residents’ subjective well-being, and provides robust empirical evidence to support previous research on the positive relationship between governance quality and subjective well-being. Finally, this study sheds light on the mechanisms through which governance quality impacts residents’ subjective well-being, providing insights into the sequential nature of the mediating mechanisms involved, and contributing to the growing body of evidence on the relationship between perceived social fairness and trust in government.

The paper is organized as follows: Section “Literature review and hypotheses” provides a literature review and develops the hypotheses to be tested, Section “Methodology” outlines the methodology, Section “Results” presents the empirical results, Section “Discussion” discusses the findings, and finally, Section “Conclusions” presents the conclusions.

## Literature review and hypotheses

Subjective well-being can be explained from two perspectives: internal characteristics (e.g., personality traits, culture, demographics) and external circumstances (e.g., income, health, environment)^16^. High-quality governance can enhance subjective well-being by improving living conditions and social welfare^3,14^. However, further empirical investigation is needed to understand the underlying mechanisms and to provide theoretical support for interventions that promote subjective well-being.

### Governance quality and subjective well-being

Subjective well-being is a psychological construct that encompasses an individual’s personal evaluation of their quality of life, including cognitive appraisals such as their satisfaction with life and emotional reactions to ongoing experiences, including positive and negative affective states^1,21^.

Governance encompasses the traditions and institutions that exercise authority within a country^7^. Governance quality refers to the degree to which public goods and services are efficiently and effectively delivered^19^. It is often assessed using composite indicators from the World Bank Worldwide Governance Indicators project, including voice and accountability, political stability and the absence of violence/terrorism, government effectiveness, regulatory quality, rule of law, and control of corruption^7^, which are widely considered reliable measures of national governance quality^19^.

Governance quality plays a crucial role in shaping citizens’ subjective well-being^4^. The literature provides valuable insights into this relationship. Consistent research findings indicate that individuals’ reported subjective well-being tends to increase as governance quality increases^15,18^. This suggests that higher governance quality contributes to a positive socio-political environment, which enhances overall subjective well-being among citizens. Transparency emerges as a key aspect of governance quality that strongly influences residents’ subjective well-being^3^ by fostering trust, accountability, and public participation. When citizens have access to information and feel that their voices are heard, they experience a sense of empowerment, leading to increased happiness and life satisfaction. Furthermore, the impact of governance quality on subjective well-being can be understood through its role in addressing corruption. Corruption erodes trust in institutions, distorts resource allocation, and ultimately leads to inequalities and decreased subjective well-being^22^. Conversely, higher governance quality actively combats corruption and ensures the fair distribution of resources, strengthens social cohesion, and promotes economic growth^17^. These factors positively influence citizens’ subjective well-being by establishing a stable and inclusive environment that supports their material and social needs.

#### Hypothesis 1

Governance quality positively influences citizens’ subjective well-being.

### The mediating effect of perceived social fairness

Humans have an inherent inclination toward fairness^23^. Social fairness encompasses the concept of social fairness in society, and involves judgments about what is considered “right” or “fair” in the distribution of resources^24^. Perceived social fairness reflects an individual’s perception of fairness in various social domains such as wealth and income distribution, work and employment opportunities, the rights and treatment of urban and rural residents, and pension and social security benefits^8^. Studies have highlighted the significant role of governance institutions in ensuring the fair exercise of public power and equitable enforcement of laws, thus establishing a positive link between governance quality and perceived social fairness^9,24,25^. The perception of social fairness influences people’s social cognition and judgment, and subsequently their behavior and subjective well-being^10,26^.

Subjective well-being is affected by a person’s emotions^1^. Drawing inspiration from Adams’s^27^ seminal equity theory, which suggests that individuals experience positive affect in response to fairness and negative affect in response to unfairness, we hypothesize that these emotional responses may lead to changes in people’s subjective well-being. Empirical studies have also shown that high-quality governance can alleviate multidimensional poverty, thereby improving social inequality^11^. Social inequality, in turn, can exert a significant influence on subjective well-being through the mediating effect of perceived social fairness^28^. Moreover, high-quality governance involves the fair delivery of public goods and services, which ultimately enhances the welfare and subjective well-being of citizens^19^. Based on these insights, we propose the following research hypothesis:

#### Hypothesis 2

Perceived social fairness mediates the relationship between governance quality and subjective well-being.

### The mediating effect of trust in government

Trust in government is a crucial component of diffuse regime support and reflects the belief that institutions will perform satisfactorily^12^. Strong trust in government serves as a valuable resource for a thriving democratic polity, reducing policy enforcement costs and generating public support for sustainable change^29^. Previous research has emphasized the role of governance quality in fostering trust in government^30^. Elements such as the effective rule of law, integrity, transparency, and accountability in public administration are fundamental to high-quality governance practices that strengthen trust in government^31^. Moreover, transparent practices and high governance quality provide opportunities for citizen participation, feedback, and expression of opinions, leading to a positive attitude toward the government^32^. Additionally, previous studies^13–15^ have established the positive impacts of trust in government on various aspects of well-being, including personal subjective well-being, which provides a solid foundation for exploring the mediating role of trust in government in the relationship between governance quality and subjective well-being. Therefore, we derive the following research hypothesis:

#### Hypothesis 3

Trust in government mediates the relationship between governance quality and subjective well-being.

### The serial mediation effects of perceived social fairness and trust in government

Fairness and trust are crucial elements in societal development, and their synergistic interplay can foster a well-organized and harmonious social order^14^. Citizens’ daily lives are closely intertwined with the distribution of social resources, and unequal distribution can result in unmet expectations among residents. If individuals perceive social unfairness and find the government’s management of social equity incongruous with their expectations, it may erode their trust in government^33^. Conversely, when the government adheres to fair procedures and equitably distributes public resources, citizens are more likely to perceive it as legitimate and trustworthy, significantly enhancing trust in government^34^. The relationship between perceived social fairness and trust in government has been elucidated in the literature. For instance, Lee^20^ revealed a positive correlation between equity in administrative processes and trust in the judiciary and law enforcement. This perspective expands our analysis beyond simple mediation effects and contributes to a more comprehensive understanding of the dynamics between governance, fairness, trust, and subjective well-being.

Governance, fairness, and trust are pivotal components of a political system and thus have profound implications for individuals’ well-being. High-quality governance, characterized by measures such as multidimensional poverty reduction and economic growth promotion, can effectively promote equity and enhance perceived social fairness^35^, foster trust in government^36^, and ultimately enhance subjective well-being by creating a conducive social environment^4,8^. Conversely, low governance quality may weaken the social fairness environment and lead to reduced perceived social fairness among individuals, which may trigger blame and hostility, further eroding trust in government and ultimately negatively impacting residents’ subjective well-being. These empirical findings underscore the significant interplay among governance quality, perceived social fairness, trust in government, and subjective well-being. Therefore, we propose a serial mediation model that elucidates the complex dynamics among these variables and their interrelationships.

#### Hypothesis 4

Perceived social fairness and trust in government sequentially mediate the relationship between governance quality and subjective well-being.

### Current study

**Figure 1:**
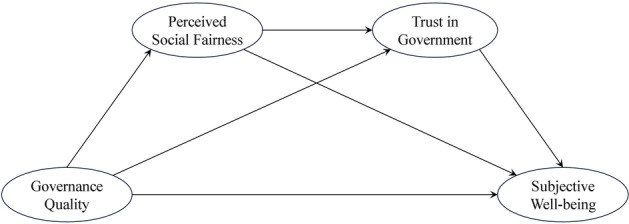
Hypothesized research model.

Based on the literature review, the objective of the present study was to investigate the relationship between governance quality and subjective well-being and its underlying mechanism. Specifically, we aimed to examine whether the relationship between governance quality and subjective well-being was mediated by perceived social fairness and trust in government. To achieve these objectives, we constructed a serial mediation model comprising subjective well-being as the endogenous variable, governance quality as the exogenous variable, and perceived social fairness and trust in government as mediators. The proposed conceptual model is illustrated in Fig. 1.

## Methodology

### Data

The data were derived from the CSS 2021, a nationally representative survey conducted by the prestigious Institute of Sociology at the Chinese Academy of Social Sciences. Initiated in 2005, the CSS aims to provide comprehensive data on Chinese families, capturing their socio-economic dynamics and revealing the challenges they faced during China’s rapid development. The CSS data serve as a vital source for understanding the socio-economic landscape of contemporary China and have been used to examine various Chinese social issues, such as happiness^37^, social fairness^38^, and trust in government^8^, among others. Thus, the CSS contributes to informed social science research and informs evidence-based policy-making. We used the most recently published data, from the 2021 survey.

The CSS 2021 consisted of a structured questionnaire administered via face-to-face household interviews. A multistage, stratified, random sampling approach was used to select participants from 30 provinces and 592 villages across China (excluding Xinjiang, Hong Kong, Macao, and Taiwan). The CSS 2021 divided the questionnaires into two categories, A and B, and randomly distributed them to 10,268 respondents. After the questionnaires were returned, the data were cleaned and a total of 10,136 responses were retained. The data used in this study came from Sample A, which consisted of 5,019 respondents. There were no missing data for the variables of interest.

In terms of demographics, the sample was composed of individuals aged between 18 and 69 years (M = 46.214, SD = 14.537), of whom 45% (*n* = 2264) were male and 55% (*n* = 2755) were female. Approximately 65% (*n* = 3274) of the respondents were rural residents and 35% (*n* = 1744) were urban residents. In terms of education, 80% (*n* = 3993) of the respondents had completed senior middle school or lower, while 20% (*n* = 1022) had completed junior college education or higher. Finally, in terms of marital status, 78% (*n* = 3913) of the respondents were married or cohabiting, and the remaining 22% (*n* = 1105) were unmarried, divorced, or widowed.

Detailed descriptive statistics of the research sample are presented in Table 1.

**Table 1:** Characteristics of the sample.

Demographics	Frequency (N)	Percentage (%)
Age		
18–30	904	18.012
31–40	894	17.812
41–50	976	19.446
51–60	1276	25.423
61 and above	969	19.307
Gender		
Male	2264	45.109
Female	2755	54.891
Hukou		
Rural	3274	65.232
Urban	1744	34.748
Missing	1	0.020
Education level completed		
Illiterate and primary school	1435	28.614
Junior middle school	1611	32.098
Senior middle school, technical secondary school, and vocational high school	947	18.868
Junior college	450	8.966
Undergraduate college	511	10.181
Postgraduate education	61	1.215
Missing	4	0.080
Marital status		
Married	3913	77.964
Other	1105	22.016
Missing	1	0.020

*n = 5019.

### Variables

#### Endogenous variable

The endogenous variable in this study was subjective well-being, which is defined as satisfaction with life. Following previous studies^39^, subjective well-being was measured using the Personal Well-being Index for Adults^40^. The survey used a 1–10 response scale to assess respondents’ satisfaction with five life domains: leisure, social life, education level, family economic status, and overall life. These five indicators were combined to form a single latent variable with high internal consistency (Cronbach’s α = 0.831).

#### Exogenous variable

The study’s exogenous variable was governance quality. The CSS 2021 survey used measurement items similar to those used in the World Bank Worldwide Governance Indicators project to assess six areas of governance quality: responsiveness, transparency, fighting crime, combating corruption, the rule of law, and guaranteeing political rights^7^. Similar definitions and measurement tools are commonly used in academic research^41^. The respondents rated these areas using a 5-point Likert scale, with 1 indicating very bad and 5 indicating very good. The six indicators were combined into a single latent variable with high internal consistency (Cronbach’s α = 0.875).

#### Mediator variables

Two mediator variables, perceived social fairness and trust in government, were included in the study. Similar to previous studies^39^, perceived social fairness is defined as an individual’s evaluation of the degree of fairness in the social distribution of resources within a particular aspect of society. It was measured by asking the respondents to rate the fairness of various aspects of wealth and income distribution, work and employment opportunities, the rights and treatment of urban and rural residents, and pension and social security benefits on a 5-point Likert scale ranging from very unfair to very fair. The four indicators were combined into a single latent variable with good internal consistency (Cronbach’s α = 0.790).

Following previous research^8^, trust in government consists of citizens’ confidence in the central government, local governments, or institutions representing government authority. To measure trust in government, the respondents were asked to rate their trust in the county government, township government, police department, and the court on a 5-point Likert scale ranging from very distrustful to very trustworthy. The four indicators were combined into a single latent variable with high internal consistency (Cronbach’s α = 0.838). The variables and their corresponding indicators are summarized in Table 2.

**Table 2:** Descriptive statistics of the survey questions.

Constructs	Indicators	Mean	SD	Range	Cronbach’s α	References
Subjective well-being	Leisure life	5.650	2.900	1–10	0.831	Herrera et al.^40^
	Social life	6.630	2.639			
	Education level	5.860	2.758			
	Economic status	6.080	2.489			
	Overall life	7.320	2.216			
Trust in government	Township	3.661	1.249	1–5	0.838	Zhi et al.^8^
	County	3.903	1.115			
	Court	3.903	1.022			
	Police	4.005	1.015			
Perceived social fairness	Urban and rural	3.069	1.245	1–5	0.771	Li and He^39^
	Wealth and income	3.186	1.209			
	Social security benefits	3.489	1.173			
	Employment opportunities	3.421	1.085			
Governance quality	Responsiveness	3.615	1.086	1–5	0.875	Mansoor^41^
	Transparency	3.681	1.054			
	Fighting crime	4.119	0.808			
	Combating corruption	3.709	1.061			
	Rule of law	3.817	0.989			
	Guaranteeing political rights	3.873	0.904			

*n = 5019.

### Statistical analysis

Statistical analyses were performed using SPSS 27.0 and AMOS 27.0. First, descriptive analyses were performed on the demographic information and research variables. Pearson’s correlation analysis was then used to assess the associations among the key variables. Confirmatory factor analysis was used to evaluate the validity and reliability of the study’s measurement instruments. Confirmatory factor analysis allowed for the construction of four latent variables: subjective well-being, trust in government, perceived social fairness, and governance quality, with factor loadings representing the relationships between the factor indicators and the latent variables. Finally, structural equation modeling was used to conduct the path analysis and test our hypotheses. Structural equation modeling is a powerful tool for investigating relationships among variables and uncovering underlying structures by testing all variables simultaneously. Furthermore, structural equation modeling accounts for measurement errors in the model and evaluates model fit using various indexes.

We used the bootstrap method as a robust approach for capturing the complexities of the mediation cases, given the limitations of traditional techniques such as the Sobel test and causal step method^42–44^. The bootstrap method was chosen for several key reasons. First, it overcomes data distribution assumptions by using resampling and estimation with 5,000 bootstrap samples, thus allowing for accurate estimates of the mediation effects. Second, the resampling nature of the bootstrap method, along with its robust estimation techniques and reliable confidence intervals (CIs), enhances the validity and reliability of the findings. Additionally, we opted for maximum likelihood estimation to ensure precise parameter estimation based on observed data likelihood, thus strengthening the analytical rigor of our study. To assess statistical significance, we examined the 95% bias-corrected and percentile CIs, thus providing dependable estimates for a comprehensive evaluation of the significance of the mediation effects.

## Results

### Descriptive statistics and correlation analysis

Table 3 presents the descriptive statistics and correlation coefficients of the study variables. The analysis revealed significant positive correlations among the latent variables, providing initial support for the study hypotheses.

**Table 3:** Means, standard deviations, and correlation coefficients.

Constructs	Mean	SD	1	2	3	4
Subjective well-being	6.308	2.018	1			
Trust in government	3.868	0.905	0.275***	1		
Perceived social fairness	3.291	0.908	0.300***	0.442***	1	
Governance quality	3.802	0.775	0.249***	0.573***	0.468***	1

*n = 5019; *SD* Standard deviation; all bivariate correlation coefficients were significant; ***p < 0.001 level (two tailed).

### Confirmatory factor analysis

The validity of the measurement models was assessed using confirmatory factor analysis. Composite reliability and average variance extracted values were used to assess the reliability and convergent validity of the constructs. The factor loadings for all items were significant at the 0.001 level, indicating good convergent validity. The composite reliability values were all above 0.7 and the average variance extracted values above 0.4, indicating acceptable reliability of the constructs^41^. These results indicate acceptable convergent validity for the measurement of governance quality, perceived social fairness, trust in government, and subjective well-being and provide support for the hypothesized model. The results are presented in Table 4.

**Table 4:** Confirmatory factor analysis results.

Constructs	Indicators	Coefficient	SE	Z	*P*	Factor loading	CR	AVE
Subjective well-being	Leisure life	1.000				0.764	0.835	0.505
	Social life	0.815	0.018	45.443	***	0.684		
	Education level	0.809	0.019	43.140	***	0.650		
	Economic status	0.782	0.017	46.226	***	0.696		
	Overall life	0.752	0.015	49.709	***	0.751		
Trust in government	Township	1.000				0.848	0.835	0.563
	County	0.885	0.013	65.614	***	0.841		
	Court	0.612	0.013	46.851	***	0.635		
	Police	0.622	0.013	48.189	***	0.650		
Perceived social fairness	Urban and rural	1.000				0.724	0.772	0.459
	Wealth and income	0.927	0.023	41.011	***	0.691		
	Social security benefits	0.881	0.022	40.350	***	0.676		
	Employment opportunities	0.740	0.020	37.250	***	0.614		
Governance quality	Responsiveness	1.000				0.750	0.876	0.542
	Transparency	0.966	0.019	52.191	***	0.746		
	Fighting crime	0.607	0.014	42.197	***	0.611		
	Combating corruption	1.019	0.019	54.835	***	0.782		
	Rule of law	0.985	0.017	57.009	***	0.811		
	Guaranteeing political rights	0.778	0.016	48.807	***	0.701		

^a^*SE* Standard error; *CR* Composite reliability; *AVE*: Average variance extracted; ***p < 0.001.

### The serial mediation effects of perceived social fairness and trust in government

Structural equation modeling was performed to assess the hypothesized model. The estimation included 5,000 bootstrap samples and the maximum likelihood estimation method was applied based on the 95% bias-corrected and percentile CIs. If the 95% CIs of the indices and indirect effects did not include zero, the mediating effect was considered statistically significant. The overall model fit indices demonstrated an acceptable fit (χ^2^ = 4,438.997, df = 146, *p* < 0.001, χ^2^/df = 30.404, root mean square error of approximation = 0.077, standardized root mean square residual = 0.041, goodness-of-fit index = 0.913, comparative fit index = 0.901).

The findings revealed that governance quality had a statistically significant positive total effect on subjective well-being (β = 0.813, *p* < 0.001), supporting Hypothesis 1. Additionally, governance quality had a significant positive effect on perceived social fairness (β = 0.626, *p* < 0.001) and perceived social fairness had a significant positive effect on subjective well-being (β = 0.670, *p* < 0.001). The specific indirect effect of governance quality on subjective well-being via perceived social fairness was also statistically significant (β = 0.419, *p* < 0.001), supporting Hypothesis 2. Similarly, governance quality had a significant positive effect on trust in government (β = 0.687, *p* < 0.001) and trust in government had a significant positive effect on subjective well-being (β = 0.335, *p* < 0.001). The specific indirect effect of governance quality on subjective well-being via trust in government was also statistically significant (β = 0.230, *p* < 0.001), supporting Hypothesis 3. Furthermore, perceived social fairness had a significant positive effect on trust in government (β = 0.282, *p* < 0.001), and the serial indirect effects of perceived social fairness and trust in government on the relationship between governance quality and subjective well-being were also statistically significant (β = 0.059, *p* < 0.001), supporting Hypothesis 4. Last, the direct effect of governance quality on subjective well-being was no longer significant when the two mediators were included in the model (β = 0.104, *p* > 0.05, 95% CI including 0), indicating that the effect of governance quality on subjective well-being was fully mediated by perceived social fairness, trust in government, and their serial mediation effects. This implies that the direct effect of governance quality on subjective well-being competes with the serial mediating effects of perceived social fairness and trust in government on the relationship between governance quality and subjective well-being. The results suggest that the positive effects of governance quality on subjective well-being need to be realized through perceived social fairness and trust in government and their serial mediating effects rather than their direct effects. The detailed results of the path analysis are presented in Table 5.

**Table 5:** The serial mediation effect of perceived social fairness and trust in government on the relationship between governance quality and subjective well-being.

Path	Point estimate	Product of coefficients		Bootstrapping					
				Bias-corrected 95% CI			Percentile 95% CI		
		SE	Z	Lower	Upper	*P*	Lower	Upper	*P*
Total effects									
Governance quality → Subjective well-being	0.813	0.047	17.298	0.721	0.910	0.000	0.720	0.908	0.000
Direct effects									
Governance quality → Subjective well-being	0.104	0.071	1.465	− 0.039	0.241	0.148	− 0.039	0.241	0.146
Governance quality → Trust in government	0.687	0.027	25.444	0.633	0.739	0.000	0.634	0.740	0.000
Governance quality → Perceived social fairness	0.626	0.020	31.300	0.588	0.667	0.000	0.587	0.666	0.000
Trust in government → Subjective well-being	0.335	0.053	6.321	0.235	0.442	0.000	0.234	0.441	0.000
Perceived social fairness → Subjective well-being	0.670	0.058	11.552	0.558	0.784	0.000	0.558	0.784	0.000
Perceived social fairness → Trust in government	0.282	0.024	11.750	0.236	0.329	0.000	0.236	0.328	0.000
Indirect effects									
Governance quality → Subjective well-being	0.709	0.052	13.635	0.610	0.815	0.000	0.609	0.813	0.000
Specific indirect effects									
Governance quality → Perceived social fairness → Subjective well-being	0.419	0.038	11.026	0.344	0.493	0.000	0.344	0.493	0.000
Governance quality → Trust in government → Subjective well-being	0.230	0.038	6.053	0.160	0.309	0.000	0.159	0.308	0.000
Serial indirect effects									
Governance quality → Perceived social fairness → Trust in government → Subjective well-being	0.059	0.010	5.900	0.041	0.081	0.000	0.040	0.080	0.000

^a^Unstandardized estimates of 5000 bootstrap samples.

**Figure 2:**
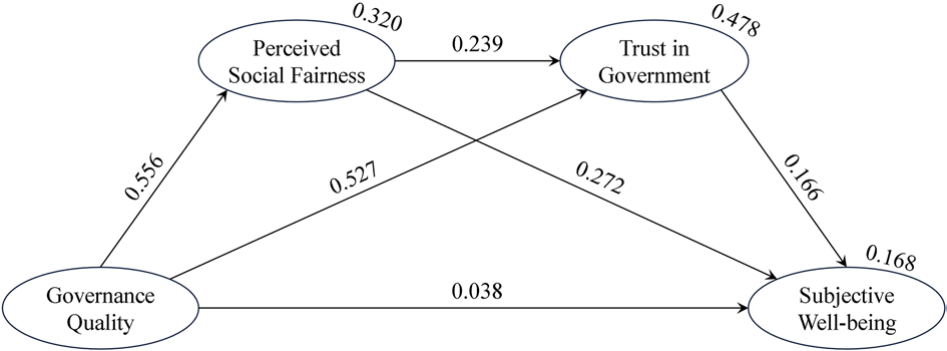
The serial mediation model of governance quality, perceived social fairness, trust in government, and subjective well-being. *Note*: Standardized estimates are shown next to the arrows. Estimates of squared multiple correlations are shown above the explained variables. Indicators of latent constructs and error/residual terms of endogenous variables are not exhibited for the sake of clarity.

Figure 2 illustrates the standardized parameter estimates derived from the serial mediation model aimed at elucidating the relationships among governance quality, perceived social fairness, trust in government, and subjective well-being. The model accounted for 32% of the variance in perceived social fairness (*p* < 0.001), 48% of the variance in trust in government (*p* < 0.001), and 17% of the variance in subjective well-being (*p* < 0.001).

Table 6 provides a concise summary of the outcomes of the hypothesis testing.

**Table 6:** Results of hypothesis testing.

Hypotheses	Results
H1: Governance quality positively influences citizens’ subjective well-being	Supported
H2: Perceived social fairness mediates the relationship between governance quality and subjective well-being	Supported
H3: Trust in government mediates the relationship between governance quality and subjective well-being	Supported
H4: Perceived social fairness and trust in government sequentially mediate the relationship between governance quality and subjective well-being	Supported

## Discussion

The results provide robust support for all of the hypotheses, revealing that governance quality positively influences subjective well-being through the individual and serial mediating effects of perceived social fairness and trust in government. These findings offer novel insights into the intricate relationship between governance quality and subjective well-being, advancing our understanding of this complex interplay.

First, our study confirms the positive influence of governance quality on subjective well-being, consistent with previous research findings^15^. In the context of China, the government has a substantial impact on people’s quality of life, which can be significantly enhanced by the implementation of public policies^14^. Globally, it is widely recognized that governments have the capacity to provide favorable institutional conditions for citizens’ happiness, and high governance quality is essential in promoting their subjective well-being^4,6^. High-quality governance, characterized by transparency, accountability, effective regulation, corruption prevention, adherence to the rule of law, and maintenance of social security, establishes a favorable institutional and social environment for individuals, thus playing a prominent role in improving their subjective well-being.

Second, our results indicate that perceived social fairness acts as a mediator between governance quality and individuals’ subjective well-being. This finding can be explained by the emergence of social inequalities in Chinese society, such as class polarization and widening wealth gaps. Given the inability of individuals to address these inequalities themselves, a high-quality governance system is essential to ensure fairness in the governing process and facilitate effective macroeconomic moderation, which helps to rectify social inequities and promote overall societal fairness. As a result, governance quality strongly influences individuals perceived social fairness and fosters their sense of belonging and identification with society. Moreover, the perception of social fairness generates hope for future development, which contributes to individuals’ subjective well-being. These findings align with those of previous studies highlighting the interconnectedness of governance quality, perceived social fairness, and subjective well-being^10,45^. Previous studies have shown that high-quality governance practices, such as reducing poverty and inequality, enhance perceived social fairness and ultimately improve subjective well-being^11,28^. Conversely, low governance quality can significantly diminish citizens perceived social fairness, leading to complaints and reduced subjective well-being. Therefore, our findings support the indirect effect of governance quality on subjective well-being through the mediating role of perceived social fairness.

Third, our findings confirm that governance quality exerts an indirect influence on individuals’ subjective well-being through the mediating effect of trust in government. This result aligns with previous research highlighting positive associations between governance quality and trust in government^12,40^ as well as trust in government and subjective well-being^13^. According to the institutional theory of government trust, the formation of trust in government is based on the actual performance of trusted entities, in which governance quality is a key factor. A high-quality governance system can effectively regulate society, combat corruption and crime within the framework of the law, and provide a secure and well-ordered social environment and society. These factors contribute to increased trust in government and foster overall social trust. Trust, as an essential component of social capital^14^, reduces defensiveness and stress, creating a less risky social environment that enables individuals to live more authentic, relaxed, and happy lives^46^. Consequently, trust in government has a noticeable positive impact on subjective well-being and is a mediating factor in the positive relationship between governance quality and subjective well-being.

Fourth, our findings reveal that the relationship between governance quality and subjective well-being is mediated by the sequential effects of perceived social fairness and trust in government. The perception of social fairness fosters trust in government, consistent with previous research^6,8,20^. This can be attributed to the cultivation of positive attitudes such as trust within a fair and just context. The perception of social fairness encourages individuals to let go of their defensiveness, promotes friendliness, and reduces discrimination, prejudice, and societal conflicts^47^. Conversely, when governance processes and outcomes are perceived as unfair, doubts may arise regarding the motives and capabilities of the governing authorities, leading to complaints, dissatisfaction, and distrust in the government. Thus, our study confirms a correlation between perceived social fairness and trust in government.

Finally, we used path analysis to investigate the sequential mediation effects of two key variables, perceived social fairness and trust in government, thus shedding new light on the connection between governance quality and subjective well-being. Our findings show that the correlation between governance quality and residents’ subjective well-being largely dissipates due to the mediating effects of perceived social fairness and trust in government. Our findings suggest that the influence of governance quality on subjective well-being operates indirectly through these intermediate factors, revealing the presence of nuanced underlying mechanisms. Our findings are in contrast to those of previous studies conducted by Helliwell et al.^4^ and Peiró-Palomino et al.,^6^ which may be attributable to our use of path analysis and country-specific factors. Path analysis allowed us to gain insights into the sequential mediation effects of governance quality, perceived social fairness, trust in government, and subjective well-being, overcoming the limitations of simple regression analysis. The reasons for the less direct correlation between governance quality and subjective well-being may be as follows. Differences between our findings and those of previous studies can be explained by contextual variations across countries or regions. In countries with well-established governance practices and strong institutional mechanisms, the direct impact of governance quality on subjective well-being may be more pronounced, aligning with the findings of Helliwell et al.^4^ and Peiró-Palomino et al.^6^. However, governance quality may affect subjective well-being in various ways, although the fairness and trust channels are the most important, depending on a country’s stage of development. In countries undergoing transitional periods or socio-political changes, the mediating roles of perceived social fairness and trust in government may become more prominent, as individuals rely on these intermediate factors to perceive and evaluate governance quality, thus influencing their subjective well-being. Cultural factors also shape the relationships between governance quality, perceived social fairness, trust in government, and subjective well-being. For instance, in collectivist societies that value social cohesion and harmony, perceived social fairness and trust in government may act as important mediators, translating governance quality into subjective well-being. Conversely, in individualistic cultures that emphasize personal autonomy and achievement, the direct impact of governance quality on subjective well-being may be more important. By thoroughly examining these contextual factors, our study offers a comprehensive understanding of how governance quality affects subjective well-being. It underscores the importance of considering governance practices, institutional mechanisms, and cultural dimensions when exploring the relationship between governance quality and subjective well-being. These insights can guide policymakers and organizations in tailoring governance strategies to promote well-being in diverse contexts.

## Conclusions

Building on the existing literature on governance quality, perceived social fairness, trust in government, and subjective well-being, this study elucidates the mediating role of perceived social fairness and trust in government in the association between governance quality and subjective well-being, both separately and serially, by conducting structural equation modeling with a sizable sample of 5019 Chinese residents. The findings provide a robust basis for future investigations aiming to elucidate the precise mechanisms underlying the association between governance quality and subjective well-being. Specifically, the results suggest that governance quality can bolster subjective well-being by fostering perceived social fairness and strengthening trust in government. Furthermore, this study enhances our theoretical and practical understanding of the impact of governance quality on residents’ subjective well-being in China.

The theoretical contributions of this study are multifaceted and substantial. First, this study extends the literature on the relationship between governance quality and residents’ subjective well-being by investigating this association within the unique context of China, which has distinct cultural, social, and political characteristics. It therefore provides valuable insights into the generalizability of governance quality theory beyond Western contexts. Second, this study proposes a novel mechanism through which governance quality influences subjective well-being by revealing the serial mediation effect of perceived social fairness and trust in government. It therefore provides a unique perspective on the underlying mechanisms through which governance quality impacts subjective well-being and advances our understanding of the complex relationship between governance, fairness, trust, and well-being.

This study also provides valuable insights for policymakers regarding the potential mechanisms through which governance interventions can impact individuals’ well-being. First, the government should prioritize improving governance quality through measures such as enhancing transparency and responsiveness, implementing more effective supervision processes to ensure the fairness of the processes and outcomes of governance, cracking down on corruption and crime, and implementing better security measures to increase people’s safety. Public governance behaviors, including governance plans, processes, and results, should be made transparent to the public to promote accountability and combat corruption. These measures are expected to enhance the public’s perceived social fairness and trust in government, consequently increasing subjective well-being. Second, social development models should prioritize equity and justice by addressing income and wealth inequality, providing basic social security to ensure a basic income for vulnerable groups and ensuring procedural fairness in policy implementation. Public resources, including education, medical care, and social security, should be distributed equitably to promote the benefits of economic and social development for all, thereby increasing residents’ perceived social fairness and enhancing subjective well-being. Third, to promote residents’ subjective well-being, the government should work on improving trust in government by empowering communities, promoting open participation in governance, and establishing open communication channels between the government and citizens. The government should demonstrate its trustworthiness to cultivate trust among those with low trust in government. Overall, enhancing governance quality is crucial for improving people’s subjective well-being, and policymakers should focus on measures that promote transparency, fairness, accountability, and trust to foster perceived social fairness and trust in government, and ultimately enhance well-being.

There are some limitations to this study that should be acknowledged. First, as the survey used in this study was cross-sectional, we cannot infer causation. Although our study is based on previous research and related theories, future longitudinal and experimental studies are needed to draw causal conclusions. Second, the study used a large, representative sample of Chinese residents, which may limit the generalizability of our findings to other countries. Additional studies are needed to test the proposed relationships in varied contexts and in comparison with our data from China. Third, the data were collected using self-report measures, which may have introduced common method bias. In future studies, governance quality ratings could be obtained from more objective sources such as professional institutes, and perceived social fairness, trust in government, and subjective well-being ratings could be obtained from respondents’ colleagues or family members to provide additional data sources for more accurate evaluation.

## Data Availability

The data were obtained from the 2021 Chinese Social Survey conducted by the Institute of Sociology of the Chinese Academy of Social Sciences. The data are available at http://css.cssn.cn/css_sy/ (in Chinese).
